# Indications for liver transplantation in adults: Recommendations of the Austrian Society for Gastroenterology and Hepatology (ÖGGH) in cooperation with the Austrian Society for Transplantation, Transfusion and Genetics (ATX)

**DOI:** 10.1007/s00508-016-1046-1

**Published:** 2016-09-02

**Authors:** Ivo Graziadei, Heinz Zoller, Peter Fickert, Stefan Schneeberger, Armin Finkenstedt, Markus Peck-Radosavljevic, Helmut Müller, Claudia Kohl, Barbara Sperner-Unterweger, Stephan Eschertzhuber, Harald Hofer, Dietmar Öfner, Herbert Tilg, Wolfgang Vogel, Michael Trauner, Gabriela Berlakovich

**Affiliations:** 1 Department of Internal Medicine, Academic Teaching Hospital Hall i.T., Milserstraße 10, 6060 Hall i.T., Austria; 2 Department of Internal Medicine II, Medical University of Innsbruck, Innsbruck, Austria; 3 Department of Gastroenterology and Hepatology, Medical University of Graz, Graz, Austria; 4 Department of Visceral, Transplant and Thorax Surgery, Medical University of Innsbruck, Innsbruck, Austria; 5 Division of Gastroenterology and Hepatology, Department of Internal Medicine III, Medical University of Vienna, Vienna, Austria; 6 Department of Transplant Surgery, Medical University of Graz, Graz, Austria; 7 Department of Psychiatry, Medical University of Innsbruck, Innsbruck, Austria; 8 Department of Anesthesiology and Intensive Care Medicine, Medical University of Innsbruck, Innsbruck, Austria; 9 Department of Internal Medicine I, Medical University of Innsbruck, Innsbruck, Austria; 10 Department for Transplantation, Medical University of Vienna, Vienna, Austria

**Keywords:** Liver cirrhosis, Acute liver failure, Hepatocellular carcinoma, Cholangiocellular carcinoma, Chronic hepatitis

## Abstract

Liver transplantation has emerged as an established and well-accepted therapeutic option for patients with acute and chronic liver failure and hepatocellular carcinoma. The disproportion between recipients and donors is still an ongoing problem that has only been solved partially over the last centuries. For several patients no life-saving organs can be distributed. Therefore, objective and internationally established recommendations regarding indication and organ allocation are imperative. The aim of this article is to establish evidence-based recommendations regarding the evaluation and assessment of adult candidates for liver transplantation. This publication is the first Austrian consensus paper issued and approved by the Austrian Society of Gastroenterology and Hepatology in cooperation with the Austrian Society of Transplantation, Infusion and Genetics.

## Introduction

Today, organ transplantation is an internationally established therapy that is indispensable in modern medicine. No other medical procedure provides a comparable improvement in quality of life. The success of organ transplantation depends to a large degree on the availability of organs. Independent of specific problems involved in the organs to be transplanted, the gap between organs needed and organs available poses a major challenge that has been only partially met over the decades. For many patients life-saving organs cannot be procured in time. For this reason, objective, internationally established and evaluated recommendations for the indication for transplantation and for organ allocation are imperative.

The goal of this work is to draw up evidence-based recommendations for establishing the indication for liver transplantation in order to help physicians manage patients who are potential candidates for a liver transplant. For the evaluation of evidence and the strength of the recommendations, the GRADE system was used (see Table 1; [1]).

**Table 1:** Grading of evidence and recommendation according to the GRADE system [1]

Evidence quality	Notes	Grading
High	Further research is very unlikely to change our confidence in the estimation of effect	A
Moderate	Further research is likely to have an important impact on our confidence in the estimate of effect and may change the estimate	B
Low	Further research is very unlikely to have an important impact on our confidence in the estimate of effect and is likely to change the estimate. Any change of estimate is uncertain	C
Recommendation	Notes	Grading
Strong	Factors influencing the strength of the recommendation included the quality of the evidence, presumed patient-important outcomes, and cost	1
Weak	Variability in the preferences and values, or more uncertainty. Recommendation is made with less certainty, higher cost or resource consumption	2

The orthotopic liver transplant (OLT) performed in almost all cases is usually the only curative therapeutic option for patients with acute and chronic liver failure and a hepatocellular carcinoma (HCC). Also, primarily genetic metabolic defects of the liver and the resulting complications can be cured with a liver transplantation (LT). In Austria, organs for transplantation are largely procured from brain-dead patients. Brain death is determined according to a standardized protocol issued by the Austrian Public Health Council (Oberster Sanitätsrat). Living liver donations are an alternative and in Austria are performed above all in pediatric patients.

Before the introduction of LT, patients with acute liver failure or decompensated liver cirrhosis had a poor prognosis. The last 25 years have brought decisive improvements in surgical techniques, postoperative care, immunosuppression, and management of LT patients, so that long-term survivors and the quality of life of transplanted persons have clearly increased. The recent assessment of (inter)national LT registers has shown 5‑, 10-, and 15-year survival rates of 75, 65, and 50 %, respectively, with the survival curves and the number of long-term survivors still increasing (www.eltr.org). For this reason, LT has for years been deemed an established therapy option with already more than 200,000 transplants performed worldwide. In Austria between 115 and 150 LT are performed each year (Austrian Federal Institute for Health, Annual Transplant Report; www.goeg.at).

## Indication

In patients with acute and chronic liver failure, the indication for LT should be assessed independently of etiology. The goal of LT is to prolong the patients’ lives and improve their quality of life. To do this, suitable patients must be chosen and the timing for the LT indication in the course of the patient’s liver disease must be determined. From the natural course of the liver disease, particularly liver cirrhosis, generally valid prognosis factors can be derived that are decisive for establishing the indication for LT. The prognosis for acute and chronic liver failure must be juxtaposed with the possible complications and the resulting morbidity and mortality, both in the immediate postoperative and the longer-term course following LT.

It is internationally agreed that a 1-year survival prognosis of less than 90 % is the minimum criterion for listing for LT [2, 3]. At a 1 -year survival rate of >90 % the prognosis for compensated cirrhosis is good in principle. Thus, the diagnosis of liver cirrhosis per se does not automatically mean that LT is a necessity. Numerous analyses have, however, shown that the occurrence of complications, in particular varices, variceal bleeding, ascites, spontaneous bacterial peritonitis, hepatorenal syndrome, and the usually accompanying decompensation of liver cirrhosis, is accompanied by a dramatic deterioration in the patient’s prognosis, with 1‑year survival rates dropping below 50 % [4]. This is usually associated with a worsening of the Child–Pugh Score from A to B or C. Because of subjective parameters (ascites, encephalopathy) and the so-called plateau effect, above all for bilirubin and prothrombin time (Quick test), this score has been replaced with the MELD (Model for End-stage Liver Disease) score that determines the prognosis for liver cirrhosis using bilirubin, INR, and creatinine values [5]. The MELD score originally served to assess the prognosis of patients before implantation of a transjugular intrahepatic portosystemic shunt (TIPS). After being modified, the score was assessed for determination of the prognosis of patients with liver cirrhosis [6] and since 2002 it has been used in the United States and since 2007 in most European centers not only for assessment of the indication for LT, but also for organ allocation [7, 8]. One publication reported that from a MELD score of ≥15, the risk for dying from liver cirrhosis is greater than the postoperative mortality following LT. Consequently, a MELD score of 15 was set as the minimum criterion for LT listing [3, 9].

The MELD score also has limitations and its application, whether for LT listing or organ allocation, is the subject of controversy [10, 11]. Numerous modifications, such as inclusion of the serum sodium level (MELD-Na score), age (iMELD), difference in the weighting of laboratory parameters and determination of the dynamic MELD score (delta-MELD) have been published, but have not met with general acceptance. One of the greatest limitations of the MELD score is the fact that it does not account for the complications of portal hypertension, which exert a very important influence on the prognosis of liver cirrhosis [10, 11]. The stages of liver cirrhosis proposed in 2006 ([4]; Table 2), which depend on variceal bleeding and ascites, are significant predictors in liver cirrhotic patients, particularly patients with a MELD score of <15 [12]. Thus, regardless of MELD score, when complications of liver cirrhosis occur, the indication for LT should be made and the patient evaluated for LT.

**Table 2:** Stages of liver cirrhosis according to D’Amico et al. [4]

Stage	Varices	Bleeding	Ascites	1-year Mortality (%)
1	−	−	−	1
2	+	−	−	3
3	+	+	−	15
4	±	−	+	26
5	+	+	+	57

Patients on the waiting list must be constantly monitored for LT necessity, because when therapy for complications (variceal bleeding, ascites) and/or the underlying disorder (antiviral therapy for hepatitis B and C, alcohol abstinence, steroid therapy for autoimmune hepatitis [AIH]) is successful, the patient’s health can clearly improve or recompensation be achieved.

Recommendations:

- Liver transplantation is indicated for patients with liver cirrhosis and a Child–Pugh score B/C or a MELD score of ≥15. ***(A 1)***.
- In patients with a MELD score of ≤14, the indication for LT can be decided on a patient-specific basis and from factors caused by the liver disease. ***(A 1)***.
- The underlying disorder and complications of liver cirrhosis require immediate therapy. If liver function improves, the LT indication will have to be re-evaluated. ***(A 1)***.

## Contraindications

Assessment for LT includes a series of examinations (see Table 3). The list of absolute medical and surgical contraindications is short and in some cases varies from center to center. There is no strict age limit for LT. An age of 70 years is discussed as the upper limit, but is not generally accepted internationally or in Austria. In exceptional cases and depending on the patient’s biological age, the age limit can be raised or lowered. Clear-cut contraindications (see Table 4) are a metastatic hepatocellular carcinoma or an untreated or noncontrollable systemic infection (sepsis). Patients with extrahepatic carcinomas should be relapse-free for several years following curative therapy before LT is given consideration. Hepatic metastatic neuroendocrine tumors and metastatic hemangioendotheliomas can be an exception. Complicating diseases such as COPD, pulmonary hypertension, and coronary heart disease are sometimes deemed a contraindication because they are relevant factors for surgical risk.

**Table 3:** Transplantation evaluation process

Hepatology evaluation	Definition of the severity and etiology of the liver disease and its prognosis (MELD, Child–Pugh score, portal hypertension and its complication)
Laboratory testing	Bilirubin (total and indirect), GOT (AST), GPT (AST), γGT, alkaline phosphatase, synthetic function (prothrombin time, INR, albumin), glucose, lipid and iron metabolism, renal function, thyroid parameters, viral hepatitis A–E, ceruloplasmin, alpha 1‑antitrypsin (genotyping), tumor markers, autoimmune parameters (ANA, AMA, SMA, LKM)
Hepatic imaging	Sonography with Doppler, MS-CT/dynamic MRT (exclusion or staging of HCC, splanchnic vessel evaluation)
Cardiopulmonary evaluation	Spirometry, arterial blood gases, (contrast)echocardiography, individual: stress-echocardiography, coronary CT, coronary angiography (CAG)
Psychosocial evaluation	Including assessment of alcohol and other addictions
Extrahepatic malignancies	Gastro- and colonoscopy, chest X‑ray (chest CT in case of special risk factors [e. g. nicotine]), ENT, gynecology/urology, dermatology
Infectiologic evaluation	CMV, EBV, tuberculosis screening (Interferon Gamma Release Assay, IGRA)
Anesthesiologic risk assessment	
Surgical risk assessment	

*MELD* Model for End-stage Liver Disease, *AST* Aspartat-Aminotransferase, *GOT* Glutamat-Oxalacetat-Transaminase, *ALT* Alanin-Aminotransferase, *GPT* Glutamat-Pyruvat-Transferase, *ANA* Antinuclear Antibodies, *AMA* Antimitochondrial Antibodies, *SMA* Smooth Muscle Antibodies, *LKM* Liver Kidney Microsomal Antibodies, *MS‑CT* multi-sclice Computed Tomography, *CMV* cytomegalo virus, *EBV* epstein barr virus, *MRT* (MRI) Magnetic Resonance Tomography (Imaging)

**Table 4:** Contraindications to liver transplantation

*Absolute*	Severe cardiac and/or pulmonary diseases and severe pulmonary hypertension (mPAP >45 mm Hg)
	Alcohol addiction without motivation for alcohol abstinence and untreated/ongoing substance abuse
	Hepatocellular carcinoma with extrahepatic metastases
	Current extrahepatic malignancies (eventually reevaluation after successful therapy)
	Sepsis
*Relative*	Untreated alcohol abuse and other drug-related addiction
	Cholangiocellular carcinoma
	Hepatic metastatic neuroendocrine tumors (NET), metastatic hemangioendothelioma
	Morbid obesity
	Persistent non-adherence

## Indications depending on the etiology of the liver disorder

### Acute liver failure

Acute liver failure is defined as an acute liver disorder with no pre-existing chronic liver damage; it can progress within 2–8 weeks to encephalopathy or even hepatic coma [13]. Etiologically, it is usually caused by acute viral hepatitis (generally hepatitis B, rarely hepatitis A, C or E), an acute manifestation of Wilson’s disease, Budd-Chiari syndrome, an autoimmune liver disorder or acute intoxication (drug [paracetamol], Amanita phalloides), idiosnycratic drug reactions or ischemia. In some patients the etiology of the acute liver failure cannot be determined with certainty. Very important for treatment success is early transfer to an LT center, where internationally recognized prognosis factors can be used to back up a request to be listed with Eurotransplant (ET) with “high-urgent” status [13]. After the high-urgent request has been approved by at least two independent experts, the next available suitable organ will be offered or made available. In almost all cases this ensures that a donor organ is obtained within 48 h. A request for high-urgent status can also be made to ET regardless of the prognosis factors, even though such factors contribute strongly to a positive decision.

The rate of spontaneous recovery and the therapeutic options for acute liver failure are extremely meager and the therapeutic options limited (e. g., steroids for autoimmune hepatitis, silibinin for Amanita mushroom poisoning, N‑acetylcysteine for paracetamol overdose). Thus, LT is the only curative therapeutic option for most patients. Consequently, the challenge is to establish the indication for LT at the right time, namely before serious complications and a contraindication for LT occur. On the other hand, against the background of organ shortage and the possible, potentially lethal complications in the postoperative course, unnecessary LT in patients with potentially reversible acute liver failure should be avoided whenever possible.

Recommendations:

- Patients suspected of having acute liver failure need to be transferred immediately to an LT center. ***(A 1)***.
- The indication for high-urgent LT is assessed by independent experts using internationally recognized prognosis factors. ***(A 1)***.

### Hepatocellular carcinoma

In the majority of cases, a hepatocellular carcinoma (HCC) is secondary to liver cirrhosis, which is a precancerous state for this disease. LT is the only potentially curative therapy approach since, by comparison to resection or local ablative therapy modalities, it also treats the underlying precancerous condition (liver cirrhosis). In every patient with HCC and liver cirrhosis an indication for LT should be assessed if a resection or radiofrequency ablation (RFA) cannot be performed because of the cirrhosis stage or other factors. Moreover, for patients in whom a resection or RFA is primarily possible, LT should be given consideration in light of the high relapse rate of >70 % after 5 years. An exception is posed by patients with a single HCC <2 cm and liver cirrhosis Child–Pugh A (according to the Barcelona Clinic Liver Cancer [BCLC] staging, BCLC 0). After successful treatment by means of resection and/or local ablation such patients can stall LT until a relapse occurs or the liver disease progresses.

In order to minimize the danger of an HCC relapse following LT and achieve a 5-year survival rate of more than 70 %, patients with favorable prognosis must be selected. Until now such selection was based on criteria that define the relapse risk exclusively on the basis of tumor size. The so-called Milan criteria, defined as a single focus ≤5 cm or maximum three tumors ≤3 cm and no evidence of macrovascular invasion or extrahepatic manifestation, have since their publication in 1996 set the standard for the selection of patients with HCC [14]. Numerous studies have shown that patients fulfilling these criteria have a lower risk for relapse, namely <20 %, and a survival rate comparable to that for other (benign) indications. Because of the usually compensated liver cirrhosis with MELD scores of ≤14 but the high risk for HCC progression while on the waiting list, patients who meet the Milan criteria are listed with a special MELD priority in the form of additional points.

If LT is restricted to patients who meet the Milan criteria, only relatively few patients with HCC will qualify for LT. Although the relapse risk following LT increases with the increase in tumor size, under some conditions patients, even those with more advanced tumor stages (so-called expanded criteria), can have a survival advantage:

The two expanded criteria that have been best evaluated are the so-called “up-to-7” [15] and the UCSF criteria ([16]; Table 5): patients for whom the total of the number of tumor lesion and the diameter of the largest nodule is a maximum of 7 (up-to-7) or who have a single lesion ≤6.5 cm or three nodules having a maximum of 4.5 cm and a total tumor size of ≤8 cm (all with no vascular invasion) can be transplanted with good results. Contrary to patients who meet the Milan criteria, these patients are not given MELD priority for LT listing.

**Table 5:** HCC listing criteria

Milan criteria [14]	Single HCC nodule ≤5 cm
	≤3 lesions each ≤3 cm
UCSF criteria [16]	Solitary HCC lesion ≤6.5 cm
	≤3 nodules each ≤4.5 cm with total diameter of ≤8 cm
Up to 7 criteria [15]	Sum of the size of the largest tumor (in cm) and the number of tumors <7

Patients who initially present in a tumor stage outside the generally accepted transplant criteria can be brought into a tumor stage inside the Milan or expanded criteria by means of resection or local ablative procedures. In such patients, the risk for relapse and the long-term survival are comparable to those of patients who initially presented with a tumor stage inside the Milan criteria [17, 18]. When choosing this therapeutic approach standardized protocols, ideally within clinical studies, should be used. Since, however, not only response to therapy but also the length of the tumor-free interval following therapy is decisive for the risk of recurrence following transplantation, a period of at least 3 months should elapse between resection or ablation and assessment of response [17, 19].

In addition to tumor stage, the histological tumor grading, meaning the degree of differentiation and the presence of microscopic vessel invasion, is a predictor for HCC relapse following LT. While macrovascular invasion can generally be demonstrated by CT before LT and is a contraindication for LT, tumor grading and microvascular invasion can be determined only by histology. Precisely for multiple tumor nodules, this can ultimately be determined with certainty only with an explanted specimen.

As surrogate parameters that reflect the tumor biology, response to local ablative therapies, serum alpha fetoprotein concentration and the tumor-free interval between treatment and LT can be used. Patients with complete or partial remission following RFA or transarterial (chemo-)embolization according to mRECIST criteria, long tumor-free interval following successful resection or local ablative therapy, low alpha fetoprotein concentration and histologically proven lack of microvascular invasion, also have a good long-term prognosis and comparable risk for recurrence after LT despite an advanced tumor stage outside the Milan or within expanded criteria [20–22]. Current scientific findings would lead us to conclude that these tumor-biological factors are more decisive for the risk for relapse than is tumor size alone [18].

In this connection it must also be mentioned that not only the risk for relapse should be considered as the basis for LT assessment, but the regional supply of organs should also be taken into account, because in comparison to other therapeutic procedures a survival advantage for HCC with cirrhosis is potentially also given for patients who do not meet the conventional LT criteria. Whether LT is possible for such patients is thus to be assessed on the basis of the weight of these patients in the overall collective of persons to be transplanted and the regional supply of organs.

Recommendations:

- For patients with liver cirrhosis and HCC who meet the Milan criteria and in whom contraindications (macrovascular invasion, extrahepatic metastases) can be excluded, LT should be considered as a curative therapeutic option by an interdisciplinary transplantation board. ***(A 1)***.
- For patients who do not fulfill the Milan criteria, but who fall within the expanded criteria (up-to-7 or UCSF criteria), and/or who have undergone successful downstaging, LT should also be given consideration. In contrast to patients who meet the Milan criteria, these patients do not receive additional (exceptional) MELD points. ***(B 1)***.
- Patients with HCC who do not meet the expanded criteria can, if complete remission persists for three to six months (assessed according to mRECIST), be evaluated for LT following a resection or local ablative procedure provided there is no vascular invasion or extrahepatic metastasis. ***(C 2)***.

### Cholangiocellular carcinoma

Surgical resection is the only established curative therapeutic option for patients with an intra- or extrahepatic cholangiocellular carcinoma (CCC). A possible indication for LT is discussed for patients with nonresectable, perihilar CCC (pCCC), patients with chronic liver failure following curative resection of a CCC or in the setting of primary sclerosing cholangitis (PSC) and in patients with mixed HCC/CCC tumors. In patients with intrahepatic CCC (iCCC) LT outside clinical studies must be viewed as a contraindication because of the poor outcome [23]. In a recent retrospective study, however, patients with a single iCCC of <2 cm achieved survival rates comparable to the 5‑year survival rates for HCC patients [24].

An American multicenter study conducted in selected patients with extrahepatic pCCC in the framework of a (neo)adjuvant radiochemotherapy concept achieved promising survival rates [25]. That protocol had a relatively high drop-out rate, meaning that only a small portion of the enrolled patients actually underwent LT. Predictive factors for dropout before LT were CA 19–9 > 500 U/ml, tumor size >3 cm, and a percutaneous tumor biopsy. The importance of the N0 stage for (peri)hilar CCCs with regard to post-LT survival was also demonstrated in a European study [26]. Advanced CCC (stage III/IV) remains an absolute contraindication.

For patients with intrahepatic, nonresectable CCC recommendations for LT are discussed very controversially in light of the scientific findings reported to date [27]. The indication for LT for patients with progressive liver cirrhosis following curative liver resection or locoregional therapies remains undecided. The recurrence-free interval, absence of lymph node metastases, and the initial tumor stage are presumed to be important prognostic criteria. Listing for LT, however, requires interdisciplinary consensus and must be determined on a patient-to-patient basis within a clinical observational study. The available data on transplantation in patients with mixed HCC/CCC tumors is overall very sparse. Recurrence risk and patient survival following LT in persons with mixed HCC/CCC tumors appear to correspond more closely to the results for CCC. With this histological differentiation, the indication for LT should thus be established with great caution [24].

Recommendations:

- In patients with extrahepatic (perihilar) cholangiocellular carcinomas in an early stage and who cannot undergo surgery because of the carcinoma’s anatomic localization and/or who have a decompensated liver cirrhosis LT can be considered as part of a multimodal therapy concept and in the setting of clinical studies. ***(B 1)***.
- Intrahepatic, inoperable cholangiocellular carcinomas (and mixed HCC/CCC tumors) should be considered for LT only in exceptional cases with low tumor stage (solitary lesion <2 cm), negative lymph node status, and possibly also participation in a multimodal therapeutic concept. ***(B 2)***.
- For patients with chronic liver failure following curative therapy of a CCC the indication for LT should be made on a patient-specific basis and under consideration of the relapse-free interval, the lymph node status, and the initial tumor stage. ***(C 2)***.

### Hepatitis C

Liver cirrhosis secondary to chronic hepatitis C virus (HCV) infection is in many European and American centers the most frequent, and in Austria the second-most frequent, indication for LT. The general recommendations for assessment and listing for LT are applicable for patients with chronic hepatitis C. Until recently, patients with decompensated HCV liver cirrhosis had no available medical therapeutic options. Almost all patients who were HCV-RNA-positive at the time of LT experienced a recurrent infection following LT, which in more than 80 % of the patients led to new chronic hepatitis C of the transplanted organ [28]. Moreover, numerous studies have shown that in comparison to the non-LT population a significantly accelerated infection course is accompanied in 30 % of patients by rapid progression to recurrent liver cirrhosis within 5 years after LT [29–31]. Until recently, HCV recurrence following LT posed a clinical challenge because of the small number of therapeutic options. Interferon-based therapy led to viral eradication in only about one-third of the patients and was additionally associated with serious, in some cases lethal side-effects [32, 33].

The availability of new directly acting antivirals (DAAs) caused a revolution not only in the therapy of HCV infection in general, but also before and after LT. Recent studies have shown that DAA therapy both before and after LT is safe, highly effective, and associated with cure rates of >90 % [34, 35]. For this reason, in HCV-RNA-positive patients an interferon-free therapy plan should be given consideration before LT, however no later than when the patient is listed, in order to achieve a possible recompensation, on the one hand, and, on the other hand, to prevent virus recurrence following LT. Ongoing antiviral therapy does not preclude LT.

Recommendation:

- For patients with HCV cirrhosis there are no disease-specific indication criteria for LT (see “Indication”). ***(A 1)***.

### Hepatitis B

Before the introduction of hepatitis B virus (HBV) immunoglobulins (HBIg) as a prophylaxis for recurrent HBV in 1993, HBV cirrhosis was deemed a contraindication for LT because of recurrence rates of up to 80 % and consequently significantly poorer survival rates. The additional introduction of oral antiviral drugs (nucleos[t]ide analogues) led to a further optimization of the long-term course of HBV patients. Initially, lamivudine and adefovir, and subsequently tenofovir and entecavir, which in addition to improved antiviral potency also have a high genetic barrier with regard to the development of resistant mutations, were used as prophylaxis as well as therapy for recurrent HBV.

Therapy with oral antiviral drugs with high genetic barrier should be commenced in all patients with HBV with liver cirrhosis and proven viral load (HBV-DNA) [36].

Antiviral therapy with tenofovir or alternatively entecavir is standard in patients with HBV cirrhosis [36]. The therapy should be continued as soon as possible following LT as recurrence prevention in combination with HBIg. The duration of HBIg administration is controversially discussed in the literature [37]. Recent studies have demonstrated that when prophylaxis with tenofovir/entecavir is continued, HBIg can be discontinued about 3 months after LT [38]. Also, patients who receive an antiHBc-positive organ must receive an HBV recurrence prophylaxis with a nucleos(t)ide analogue lifelong [36].

Recommendation:

- For patients with HBV cirrhosis there are no disease-specific indication criteria for LT (see “Indication”). ***(A 1)***.

### Alcoholic liver cirrhosis

Alcoholic liver cirrhosis is worldwide the second-most frequent, and in Austria the most frequent, indication for LT. For patients with alcoholic liver cirrhosis, the hepatic complication of alcoholism or of chronic alcohol abuse, psychiatric/psychological assessment plays an especially important role in the work-up. Acceptance of alcoholism by the patient and his family, social integration, as well as a stable socioeconomic situation are the *conditio sine qua non* for LT. Patients must accept their alcoholism and be willing to abstain from alcohol for their whole life as well as undergo pre- and postoperative psychiatric care and must also be socially integrated. These criteria are taken from studies that show that a positive family history with regard to alcoholism, a lack of social support, psychiatric comorbidities as well as a brief abstinence period before LT are risk factors for recurrent alcohol abuse or alcoholism ([39–42]; Table 6). The often postulated compulsory 6‑month abstinence period is, however, the subject of controversy. Abstinence before LT is absolutely recommended from a medical standpoint because of the potential for recompensation of the liver disease. However, the mentioned “6-month abstinence rule”, is arbitrary and not evidence-based. In patients who first present with severe decompensation of liver cirrhosis and in patients with acute alcoholic hepatitis, such an abstinence period would usually exclude LT as a treatment option because of the very poor short-term prognosis. In these cases psychiatric or psychosocial assessment is the key factor. A French study showed that patients with acute alcoholic hepatitis, who underwent a detailed assessment procedure with strict selection criteria, enjoyed an excellent prognosis following LT [43].

**Table 6:** Alcohol-specific risk evaluation for liver transplantation

*No/low risk patient (minimal risk for recurrence)*	Patient with alcohol abuse, good motivation for abstinence, and no risk factors^a^
*Medium-risk * *patient (medium risk for recurrence)*	Patient with alcohol abuse and poor awareness of the problem, ambivalent motivation for abstinence, and one or more risk factors^a^
	Patient with alcohol dependence, good motivation for abstinence, and one risk factor^a^
	Patient with alcohol dependence, good motivation for abstinence, long period of abstinence, and multiple risk factors^a^
*High-risk patient (high risk for recurrence)*	Patient with alcohol abuse and no motivation for abstinence
	Patient with alcohol dependence and with two or more risk factors^a^

^a^Risk factors: positive family history, poor social support, psychiatric comorbidities, short period of abstinence prior to LT

The challenge in treating patients with alcoholic liver cirrhosis lies not only in selection for LT, but especially also in the need for life-long follow-up care in order to be able to recognize any recurrent alcohol intake early in the postoperative course and to intervene at an early time in order to prevent recurrent liver disease and/or extrahepatic alcohol-associated illnesses. In the case of concomitant nicotine abuse, especially patients with alcoholic liver cirrhosis exhibit a significantly elevated risk for secondary malignancies (lung, oropharynx) following LT [44, 45].

Recommendations:

- Patients with alcoholic liver cirrhosis are subject to the generally valid indication criteria for LT, with psychiatric/psychological assessment being an especially important additional factor. ***(A 1)***.
- A high risk exhibited by patients in psychosocial assessment (see Table 6) is a contraindication. ***(B 1)***.

### Primary biliary cholangitis (PBC)

The criteria for the indication for LT do not differ importantly from other indications, and are given in the cirrhosis stage with Child–Pugh B or a MELD score >15. Moreover, in patients with serum bilirubin levels >6 mg/dl, irrespective of the Child–Pugh stage or MELD score, LT should be considered and possible accompanying causes for aggravated hyperbilirubinemia such as hypothyroidism concomitant with Hashimoto’s thyroiditis or infections must be ruled out [46]. For PBC patients with inacceptable quality of life as a result of therapy-resistant pruritus or worsening sarcopenia, the indication for LT should be established regardless of MELD score [47]. The prognosis following LT is excellent with a 5-year survival rate of >85 % [48]. The presence of a chronic fatigue syndrome alone, which is often pronounced in PBC patients, is not an indication for LT. In PBC patients attention must already be paid to the great probability of a co-existing, often severe osteoporosis, for which specific therapy is to be commenced already pre-LT. Clinically relevant coagulopathy typically occurs in PBC patients only in very advanced stages of the disease.

Recommendations:

- The indication for LT is given for PBC patients with liver cirrhosis, Child–Pugh B/C, MELD score ≥15, and serum bilirubin levels >6 mg/dl. ***(A 1)***.
- Therapy-refractory pruritus may also be an indication for LT in PBC patients. ***(B 1)***.

### Primary sclerosing cholangitis (PSC)

LT is the only curative therapy for advanced PSC with excellent 10-year survival rates of 80 % [46, 49]. In PSC patients the hepatocellular capacity to produce blood coagulation factors and serum proteins is usually sustained for a long time and complications of portal hypertension such as therapy-resistant ascites and hepatorenal syndrome often occur late in the course of the disease. Severe recurring bacterial cholangitis, progressing marasmus or therapy-resistant pruritus should cause a patient to be assessed for LT. The unique feature of this disease is also that in addition to the general criteria for advanced cholestatic cirrhosis, the criteria of a premalignant disease are also to be given consideration. Because of the high recurrent rates and the associated very poor prognosis extra- and intrahepatic CCC is only in its early stages not a contraindication for LT (see “Contraindications”). To date, there are neither special imaging procedures, serological tumor markers nor other procedures that can give reliable predictive values for the diagnosis of early CCC. PSC patients with dominant bile duct stenoses or previously diagnosed bile duct dysplasia should be immediately discussed for the possibility/necessity of LT already in a precirrhotic stage. Due to the lack of sufficient scientific data, there are currently no guidelines for the management of these complications; thus, the decision to perform a LT remains difficult and patient-specific. Because of the elevated CCC risk in PSC patients, resection of the extrahepatic bile ducts with a roux-en-Y choledochojejunostomy is the surgical procedure of choice in several centers although there is no clear evidence for the superiority of this procedure by comparison to the classical donor-to-recipient common bile duct anastomosis [50]. PSC patients with concomitant inflammatory bowel disease (PSC-IBD) should undergo a screening colonoscopy at 1‑ to 2‑year intervals before and after LT because of the substantially elevated risk of colorectal carcinoma.

Recommendation:

- In addition to decompensated cirrhosis, which in PSC patients often occurs late in the course of the disease, severe recurring cholangitis, therapy-refractory pruritus, and dominant bile duct stenosis or dysplasia can be the indication for LT, irrespective of MELD score. ***(A 1)***.

### Autoimmune hepatitis (AIH)

With 4 –6 %, AIH is a relatively rare indication for LT in European centers. LT can become necessary in AIH patients as a result of delayed or incorrect diagnosis and thus delayed commencement of immunosuppressive treatment, therapy failure or inadequate treatment compliance [51–53]. Thus, LT should be considered in AIH patients who fail to primary therapy presenting with (sub)acute liver failure or in patients with decompensated liver cirrhosis (MELD >15). Twenty-five percent of AIH patients may present with (sub)acute liver failure at first manifestation; for such patients a high-urgent listing is possible.

Recommendations:

- Patients with decompensated liver cirrhosis who do not respond to medical therapies should be assessed for LT. ***(A 1)***.
- For patients with a fulminant disease, the same recommendations pertain with regard to indication and the request for a high-urgent listing as for acute liver failure due to other causes (see “Acute liver failure”). ***(A 1)***.

### Nonalcoholic steatohepatitis (NASH)

Recent studies have shown that the incidence of non-alcoholic fatty liver cirrhosis, usually as a result of the metabolic syndrome (obesity, hyperlipidemia, diabetes mellitus, arterial hypertension), has clearly increased in recent years. In light of the dramatic increase in new cases of (morbid) obesity and diabetes mellitus, NASH could in future be the most common indication for LT [54].

Since patients with NASH cirrhosis have a clearly elevated risk for cardiovascular comorbidities, it is imperative that these patients undergo an especially thorough preoperative cardiovascular work-up. Therapeutic optimization for cardiovascular risk factors should already be performed preoperatively. Postoperatively, patients with NASH cirrhosis also show a clearly elevated risk for cardiovascular risk factors as well as for cardiovascular diseases [55]. For this reason, early postoperative diagnosis and treatment of the characteristic symptoms of metabolic syndrome, diabetes mellitus, hyperlipidemia, arterial hypertension as well as obesity are important, because immunosuppressive therapy can further increase the negative metabolic effect.

#### Bariatric surgery before/after LT

The scientific evidence for bariatric surgery before/during or after LT does not allow any clear guidelines to be established. However, the published case series permit the following conclusions to be drawn [56–59]:

- Bariatric surgery simultaneously with LT is associated with high morbidity.
- Bariatric surgery after LT is also associated with a higher risk. The high perioperative risk profile for LT remains unchanged. The indication for bariatric surgery should follow the conventional criteria for bariatric surgery and not be performed earlier than 6 months after LT.
- A sleeve gastrectomy before LT has a potentially positive influence on perioperative LT morbidity and is associated with an acceptable risk profile. The decision should be made on a patient-specific basis after having exhausted alternative possibilities.

Recommendations:

- Patients with NASH cirrhosis have no disease-specific indication criteria for LT (see “Indication”). ***(A 1)***.
- Because of the frequent association with a metabolic syndrome, special attention must be paid to cardiovascular comorbidities.*** (A 1)***.

### Inherited metabolic liver diseases

Metabolic liver diseases are a heterogeneous group of diseases that in adults include hemochromatosis, Wilson’s disease, and alpha 1‑antitrypsin deficiency as the most frequent metabolic causes of liver cirrhosis.

Although the risk for progression to liver cirrhosis as a complication of hemochromatosis can be prevented by early commencement of phlebotomy, in some patients hemochromatosis is diagnosed already in the cirrhotic stage. In these cases, hemochromatosis is diagnosed on the basis of the genetic analyses (homozygosity for C282Y in the HFE gene), as the changes in iron metabolism in the advanced stage of any cirrhosis resemble those seen in early hemochromatosis [60]. Provided there are no contraindications, therapeutic phlebotomy should also be performed in the advanced cirrhotic stage. In rare cases, phlebotomy may lead to recompensation of the cirrhosis. Hemochromatosis is also associated with a relatively high risk for the development of HCC compared to other etiologies; HCC can even occur in a non-cirrhotic liver [61]. In patients with liver cirrhosis, the indication for LT is determined by the stage of the liver disease or the occurrence of HCC. Survival following LT in patients with hemochromatosis is poorer than for other indications because of the cumulative occurrence of infectious complications [62]. Even if the metabolic deficiency of the underlying cause of hemochromatosis is eliminated with LT, hemochromatotic complications already existing before LT such as arthropathy, cardiac insufficiency of diabetes are not cured by LT alone. Another special feature of patients who are transplanted due to hemachromatosis is, compared to other indications, an elevated risk for severe infections and consequently leading to a poorer post-LT survival [63].

In addition to hemochromatosis, Wilson’s disease is another metabolic disease that can lead to liver cirrhosis and HCC [64, 65]. Similar to hemochromatosis, the indication for LT in patients with Wilson’s disease primarily depends on the stage of liver cirrhosis. Treatment of Wilson’s disease entails administration of the copper chelators trientene and D‑penicillamine, whereby commencement of therapy, which should also be started in a cirrhotic stage, can be followed by an initial deterioration of symptoms. In addition, patients with Wilson’s disease can also present with acute liver failure independent of the stage of their liver disease. For this reason and contrary to other chronic liver diseases, patients with Wilson’s disease presenting with fulminant acute liver failure in a cirrhotic stage can also be assessed for a so-called “high-urgent” listing. In addition to hepatic symptoms, patients with Wilson’s disease can also present with neurological disorders. Since LT can slow the progression of neurodegeneration, also isolated neurological symptoms of the disease are a potential LT indication [66]. The metabolic defect of Wilson’s disease, which causes copper accumulation in various organ tissues, is cured by LT, thus, eliminating the need for chelator therapy following LT.

The third metabolic liver disease encountered in adults is alpha 1‑antitrypsin deficit (A1AD). Certain mutations in the alpha 1‑antitrypsin gene cause aggregation of the misfolded protein. In part of the homozygous or compound heterozygous patients this can cause either neonatal cholestatic hepatitis or liver cirrhosis in young adults. The indication for LT in patients with liver cirrhosis caused by an underlying A1AD is established primarily by the stage of the liver disease and should be considered in patients with a MELD score ≥15 [67]. Since the deficiency of alpha 1‑antitrypsin is also associated with an increased risk for emphysema, a differentiated assessment of pulmonary function should be performed before LT. In patients with A1AD, the biochemical defect is cured by LT, which can be seen as a normalization of the alpha 1‑antitrypsin concentration in the blood following LT. Thus, LT can also prevent progression of the pulmonary disease.

Recommendations:

- In patients with liver cirrhosis associated with hemochromatosis, Wilson’s disease or alpha 1‑antitrypsin deficiency, the general indication criteria for LT apply (see “Indication”). ***(A 1)***.
- In the case of progressive neurological symptoms of Wilson’s disease despite medical therapy, LT is to be given consideration. ***(B 1)***.
- For patients with Wilson’s disease presenting with fulminant liver failure, the indications for acute liver failure apply, irrespective of the stage of the chronic liver disease (see “Acute liver failure”) ***(A 1)***.

### Rare liver diseases (indications irrespective of MELD score—“MELD exceptions”)

Rare indications for LT are primary hyperoxaluria, inherited amyloidosis, the hepatic manifestation of cystic fibrosis, polycystic liver–kidney disease and other rare genetic storage, and tumor diseases or dysplasia (such as polycystic liver disease, Osler-Weber-Rendu disease). The former of these diseases can necessitate a multiorgan transplant (combined liver–renal transplant or combined liver–heart[lung] transplant), depending on the patient’s clinical situation. Since these patients only achieve a score of >15 in exceptional cases and because the MELD and Child–Pugh scores do not reflect the actual severity of the disease, they are grouped together under the term “MELD exception indications” (see Infobox 1). Hepatopulmonary syndrome and therapy-refractory pruritus are also included in this group.

#### Infobox 1

Regarding the list of diseases, for which Eurotransplant provides “standard exceptions”, please, see Eurotransplant Manual, Chapter 5 (https://www.eurotransplant.org/)

Recommendation:

- The LT indication in patients with diseases, whose severity/prognosis is not properly reflected by the MELD or Child–Pugh score, must be assessed interdisciplinarily on an individual patient basis. ***(B 1)***.

### Liver transplantation in HIV-positive patients

Because of the great efficacy of antiretroviral therapy options for HIV infection, patients with controlled HIV infection (CD4 cell count >100/µl, nondetectable viral load) should be considered for LT [68]. In recent years HIV patients have been increasingly transplanted with success and their survival, except for those with pre-existing HCV co-infection, was identical to that for other indications. For HCV co-infected patients, new, highly potent therapeutic options (see hepatitis C) have recently become available. These permit successful virus elimination before and after LT in a majority of patients. For patients with HCV co-infection an interferon-free antiviral therapy, just as for HCV mono-infected patients before LT, is to be given consideration.

Recommendation:

- HIV-positive patients with non-detectable viral load and appropriate hepatologic indication should be assessed for LT. ***(A 1)***
